# Soft Wireless Passive Chipless Sensors for Biological Applications: A Review

**DOI:** 10.3390/bios15010006

**Published:** 2024-12-26

**Authors:** Mingguang Zhang, Mengyun Li, Wei Xu, Fan Zhang, Daojin Yao, Xiaoming Wang, Wentao Dong

**Affiliations:** 1 School of Electrical and Automation Engineering, East China Jiaotong University, Nanchang 330013, China; 2023028085406024@ecjtu.edu.cn (M.Z.); 2023028085406023@ecjtu.edu.cn (M.L.); 2022028082302002@ecjtu.edu.cn (W.X.); 3060@ecjtu.edu.cn (D.Y.); 2501@ecjtu.edu.cn (X.W.); 2 Department of Mechanical and Electrical Engineering, Guangdong Polytechnic Normal University, Guangzhou 510665, China; etuni@126.com

**Keywords:** soft sensors, wireless passive sensors, wireless passive LC-resonant sensor, surface acoustic wave, wireless radio frequency sensor, biological monitoring

## Abstract

Soft wireless passive sensors have been applied in biological, engineering, and other fields due to their advantages in powerless supply and remote data transmission. External information is obtained by soft wireless passive sensors via the external coils based on electromagnetic induction. The purpose of this review paper is to outline the biological applications of soft wireless passive chipless sensors and provide a classification of wireless passive sensors and an overall explanation of the main work. Three kinds of soft wireless sensors, soft wireless passive LC-resonant sensors, soft wireless radio frequency (RF) sensors, and soft wireless surface acoustic wave (SAW) sensors, are introduced with their working principles, equitant circuits, and biological applications. Soft wireless passive sensors with integrated LC-resonant units are applied to physical quantity measurements for denoting the mapping relationship between the frequency resonance and the monitored object. Utilizing the electromagnetic field principle, RF sensors enable wireless measurements and data exchange of physical parameters. SAW sensors with piezoelectric substrates are applied to physical parameter monitoring using guided waves in monitoring objects. Soft wireless passive sensors aim to monitor biological health without an external power supply or wired data communication, which would bring increased convenience to the lives of the people who use them.

## 1. Introduction

Soft wireless passive sensors have been applied to biological monitoring due to their advantages of powerless supply and remote data transmission [1,2,3,4]. Soft sensors with enough flexibility could be applied to soft tissues in deformation monitoring to provide more accurate health data [5,6,7,8]. Soft wireless passive sensors can work for a long time without an external power supply, and they are remotely powered by electromagnetic or acoustic wave energy exchange, utilizing electromagnetic induction, acoustic propagation, and response to environmental changes [9,10,11,12]. Soft wireless passive sensors with chipped devices affect the wearability properties for continuous biological health monitoring. It would be more demanding of the design and application of soft wireless passive chipless sensors for them to seamlessly integrate onto the skin surface for long-term biological health monitoring.

Soft wireless passive sensors could receive different signals under different excitation conditions in different application fields [13,14,15,16,17]. According to the detection principles of soft wireless passive sensors, they could convert biological signals into electrical signals [2,18]. Soft wireless passive sensors have a great advantage in their simple structure for biological health data recording [19,20,21,22]. There are many review articles that have been published devoted to research in and changes in soft wireless passive sensors [15,19,23,24,25]. The advances in the principles, design, fabrication, and applications of wearable SAW sensors for biological monitoring have been comprehensively illustrated [26]. Chipless radio frequency identification (RFID) sensors have been comprehensively analyzed for in-body implant applications [27]. This mainly focuses on the wireless chipless or chipped sensor for biological health applications. The application progress and challenges of wearable biosensors based on ultra-high-frequency RFID technology in diabetes management are summarized, and the development of continuous monitoring biosensors is discussed [28]. The similar and dissimilar properties of soft wireless passive sensors in different categories should be delineated to improve the design strategies and applications of soft sensors.

**Figure 1:**
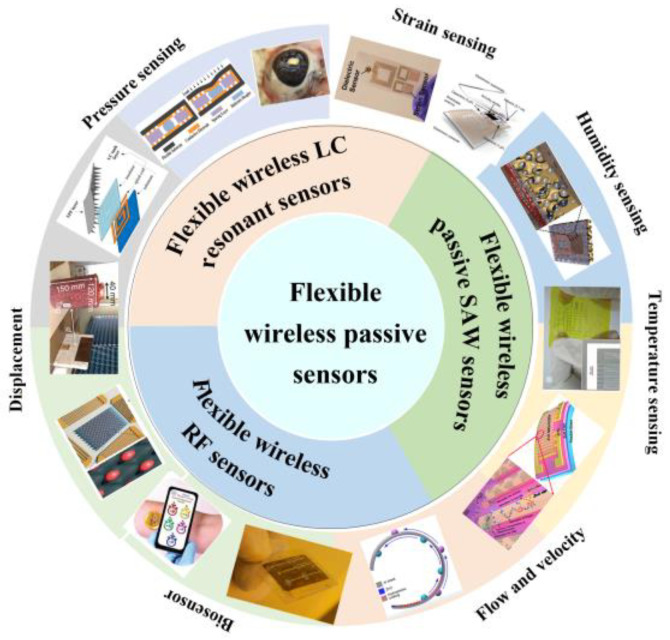
Classification and application of soft wireless passive chipless sensors for strain, pressure, temperature, flow and velocity, and humidity measurement.

This review article focuses on the principle and use in biological health monitoring of soft wireless passive chipless sensors for enhancing their softness and wearability properties. Figure 1 depicts the principles and biological applications of three kinds of soft wireless passive chipless sensors: soft wireless passive inductor–capacitor (LC) resonant sensors, soft wireless SAW sensors, and soft wireless RF sensors. (1) Soft wireless passive LC-resonant sensors rely on inductive coupling [29,30], which changes the resonance frequencies of resonant circuits with inductors and capacitors. Different external loads on the soft wireless passive sensors lead to a variable frequency shift, which could be applied to strain, pressure, and sweat monitoring for wearable electronics, human–computer interactions, and biomedical applications. (2) Soft wireless RF sensors utilize radio frequency signals for data transmission and environmental monitoring and are capable of operating within specific frequency bands [31,32]. Due to their excellent mechanical flexibility, durability, and passive wireless characteristics, they are suitable for applications in medical monitoring fields such as heart rate and blood glucose level monitoring as well as for communication between wearable devices and external systems. (3) Soft wireless SAW sensors with interdigitated transducers (IDTs) deposited on a piezoelectric material are designed for biological health monitoring due to their high sensitivity, good biocompatibility, easy integration, and miniaturization [15,33,34]. They can convert mechanical signals into electrical signals via the SAW sensor.

This research aimed to provide design strategies for and approaches for the classification of soft wireless passive sensors. The principles of three kinds of soft wireless sensors are introduced for multi-parameter perception: pressure [35,36], strain [37,38], temperature [29,39,40], flow and velocity [41,42], humidity [43,44], and biomicrofluidic monitoring [45,46] for biological applications.

**Figure 2:**
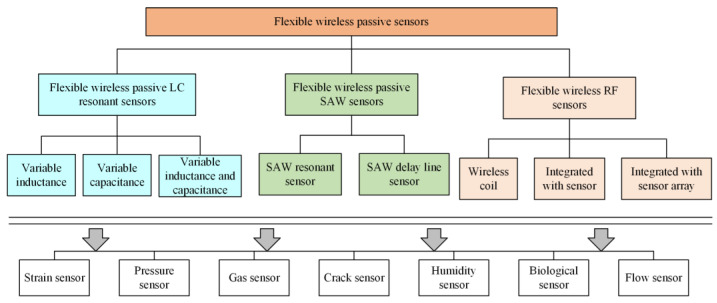
Classification and monitoring parameters of soft wireless passive chipless sensors.

Soft wireless passive chipless sensors are divided into three categories with corresponding characteristics and applications (Figure 2), which are classified by the principle based on the LC-resonant, surface acoustic wave sensing method, and radio frequency sensing technology. Soft wireless passive chipless sensors without external power supply and chipped devices are more convenient for biological health monitoring by providing novel applications under extensive attention compared to conventional rigid sensors [14,15,19,24].

(1) Soft wireless passive chipless sensors with LC-resonant circuit could applied to dielectric assessment of skin properties, hydration measurement, and skin strain [47,48]. Due to the working principles of LC-resonant circuits, the variable resonance frequency shifts with variable inductance, variable capacitance, and variable resistance value are adopted into the design and application of soft wireless passive sensors due to external excitation. External sensor readout systems with data transmission and receiving units are adapted to wearable or portable electronic applications.
(2) Wireless RF sensors are mainly composed of three parts: tags, antennas, and readers. These are able to realize remote monitoring and control by transmitting data through radio waves [49,50]. With sensitive response, low power consumption, and high biocompatibility, these have a wide range of applications in the fields of biomedical monitoring, miniature RFID tags, and wireless passive implantable electronics.
(3) SAW sensors are mainly composed of piezoelectric substrates, interdigital transducers (IDTs), and reflective gratings [15,51]. SAW sensors receive electromagnetic signals from the reader, and piezoelectric materials are used to generate acoustic waves under the excitation of electrical signals [14,52]. The resonant frequency of the SAW sensor is related to the acoustic wave propagation velocity in the medium, the geometry parameters, and the design of the electrode. SAW sensors play a crucial role in the detection of chemical, gas, and biological sensing signals, and have gained extensive attention in the field of biomedicine and microfluidics.

The design, principles, and applications of soft wireless passive chipless sensors are discussed comprehensively to determine the research tendency and future perspectives. Soft wireless passive chipless sensors are widely applied to biological health monitoring, including temperature, strain, humidity, and biosensors for human activities and body gesture monitoring. Finally, the key challenges and future perspectives of soft wireless passive chipless sensors or applications in wearable healthcare systems are discussed. The work is arranged as follows: The principles of soft wireless passive sensor with LC-resonant sensors, soft wireless SAW sensors, and soft wireless RF sensors are illustrated in Section 2. Biological applications based on soft wireless LC-resonant sensors are introduced in Section 3. Biological monitoring applications based on soft wireless RF sensors are described in Section 4. Soft wireless SAW sensors are applied to biological, wearable, and microfluidic electronics in Section 5. The whole work and future perspectives are concluded in Section 6.

## 2. Principles of Flexible Wireless Passive Sensors

External information is obtained by soft wireless passive sensors via external coils; perception and transmission information is exchanged based on electromagnetic induction [14,52]. Soft wireless passive chipless sensors can be categorized into three groups according to their schematic graphs, equitant circuits, and operation principles (Table 1): soft wireless passive LC-resonant sensors, soft wireless RF sensors, and soft wireless SAW sensors. Soft wireless passive sensors with LC-resonant units are applied in physical quantity measurement for determining the relationship between frequency resonance and monitored parameters. More details for soft wireless passive LC-resonant sensors are provided in Section 2.1. RF sensors are capable of operating across various frequency ranges, enabling the real-time measurement of multiple physical quantities through the modulation and demodulation of radio frequency signals. The working principles of soft wireless radio frequency sensors are introduced in Section 2.2. SAW sensors generate acoustic surface waves on the surfaces of piezoelectric substrates. When the acoustic waves encounter changes in the external environment (e.g., pressure, temperature), their characteristics (e.g., wave speed, frequency) are also changed. The electrical output of the SAW sensor could be applied in physical measurement detection. The working principles of soft wireless SAW sensors are detailed in Section 2.3.

### 2.1. Wireless Passive LC-Resonant Sensor

Wireless passive chipless sensors with RLC components (inductors, capacitors, and resistors) can be formed into RLC-resonant circuits [40,58]. Wireless passive sensors based on LC resonance circuits are adopted to detect external physical quantities with variable resonance frequency shifts due to the variable inductance, capacitance, and resistance values; this is represented as:

(1) $$f_{resonant}=\frac{1}{2\pi }\sqrt{\frac{1}{L_{s}C_{s}}-\frac{R_{s}^{2}}{L_{s}^{2}}}≅\frac{1}{2\pi \sqrt{L_{s}C_{s}}},R_{s}^{2}\ll \frac{L_{s}}{C_{s}}$$

where $L_{s}$ is the inductance value of RLC circuit, $C_{s}$ is the capacitance value of RLC circuit, and $f_{resonant}$ is the resonance frequency value of RLC circuit.

Wireless passive chipless sensors based on LC resonance can be applied to detect external physical quantities with the variable resonant frequency, which is divided into variable inductance, capacitance, and resistance forms. Wireless passive LC sensors with different combinations and integration methods of inductance, capacitance, and resistance are designed for strain and pressure monitoring due to their variable resonance frequency shift. Through the electromagnetic coupling effect of wireless coils, remote wireless signal acquisition is achieved for external load measurement with the resonant frequencies of an RLC circuit. The variable resistance value would lead to the quality factor changes of resonant circuits.

**Figure 3:**
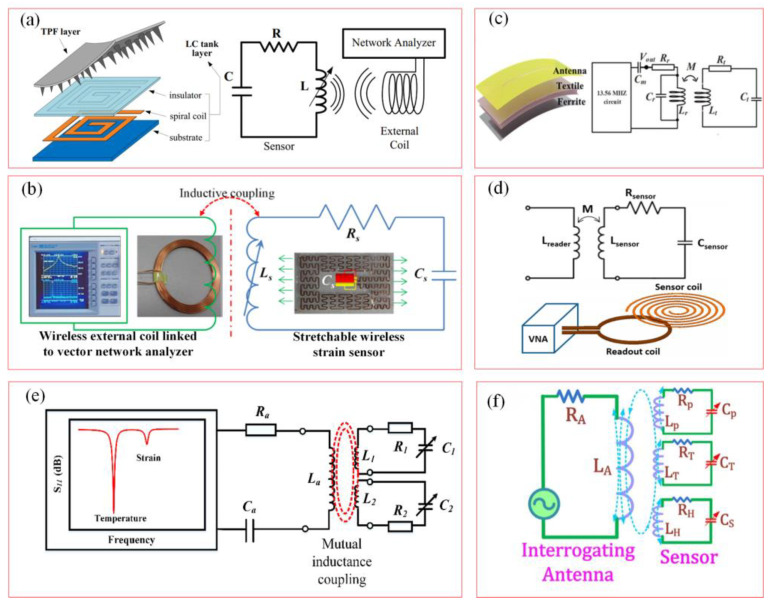
Working principles and monitoring parameter analyses of wireless passive LC-resonant sensors. (**a**,**b**) Wireless passive sensors based on changes in antenna inductance [36,38]. (Copyright (2021) IOP Publishing Ltd., Bristol, UK. Copyright (2015) Elsevier, Amsterdam, The Netherlands). (**c**) Wearable wireless pressure sensor for human motion detection [59]. (Copyright (2023) WILEY-VCH, Weinheim, Germany). (**d**) A wireless passive LC sensor system [60]. (Copyright (2023) American Chemical Society, Washington, DC, USA). (**e**,**f**) Wireless passive sensors based on multi parameter synchronous sensing with multiple capacitance variations [40,61]. (Copyright (2018) Elsevier, Amsterdam, The Netherlands).

Figure 3a,b show the working principles and equivalent circuit of the soft wireless passive LC-resonant sensor with variable inductance value [36,38]. A wireless coil system with a suitable inductance is designed for external strain and pressure monitoring, which would cause inductance variation under the external excitation. The inductance value of the wireless coil changes with the external strain, resulting in a resonant frequency shift. The variable resonant frequency shift value is collected by the vector network instrument via the variation in the external strain of the wireless coil, and it is represented as:

(2) $$f_{resonant}\left(\varepsilon \right)=\frac{1}{2\pi \sqrt{L_{s}\left(\varepsilon \right)C_{s}}}$$

where ε is the external strain of the wireless coil and $L_{s}\left(\varepsilon \right)$ is the inductance value of the RLC-resonant circuit, and it has a functional relationship with the external strain ε of the wireless coil.

Figure 3c presents a soft wireless pressure sensor based on an LC resonator with a signal reading circuit [59]. The circuit consists of a microcontroller and a Bluetooth module. The external pressure is detected by the variable resonant frequency, and the pressure-dependent resonant frequency is converted to the output voltage of the circuit to achieve high sensitivity. At the same time, Bluetooth wireless communication protocol is used to remotely transmit data to external devices. This has been applied in wearable devices and human motion detection. The resonant frequency value of the resonance circuit is collected through an external coil to achieve the external pressure measurement.

(3) $$f_{resonant}\left(\varepsilon \right)=\frac{1}{2\pi \sqrt{L_{s}C_{s}\left(p\right)}}$$

where p is the external pressure applied to the wireless coil, and $C_{s}\left(p\right)$ is the capacitance value of the RLC circuit; it has a linear relationship with the external pressure, p.

Figure 3d depicts the working principle and physical layout of the wireless passive LC sensor system [60]. The readout coil communicates with the sensor coil through electromagnetic coupling, and a vector network analyzer is used to measure and analyze the resonance characteristics of the sensors for the analysis of biomedical parameters in a closed environment species. Figure 3e shows the multi-parameter perception principle and a circuit model of a wireless passive LC sensor [61]. Figure 3f is simplified into multiple LC-resonant circuits composed of multiple inductors, capacitors, and resistors in series [40]. *L_T_*, *L_P_*, and *L_H_* represent the equivalent inductance values of temperature, pressure, and humidity sensor coils; *C_T_*, *C_P_*, and *C_H_* represent the equivalent capacitance values of temperature, pressure, and humidity sensors; *R_T_*, *R_P_*, and *R_H_* represent the equivalent resistance values of temperature, pressure, and humidity sensors; *L* represents the equivalent inductance value of the reading antenna; *R* represents the equivalent resistance value of the reading antenna. From the perspective of circuit design and sensing principles, wireless LC-resonant sensors with multi-parameter sensing abilities are synchronously applied in biological monitoring.

### 2.2. Wireless Passive RF Sensors

The soft wireless chipless RF sensor, as a novel type of sensing technology, utilizes RF signals for data acquisition and transmission. Highly sensitive materials with variable conductivity, dielectric constant, or permeability are utilized in the chipless RF sensor research area. The physical parameters of the materials change in response to external physical quantities such as temperature, humidity, and pressure, thereby altering the electromagnetic response of the sensor label. A variation in electrical conductivity leads to a corresponding change in the tag response amplitude, while modifications in dielectric constant or permeability affect the resonance frequency or phase of the sensor. The sensor data are modulated by a radio frequency signal and transmitted wirelessly for demodulation and analysis.

**Figure 4:**
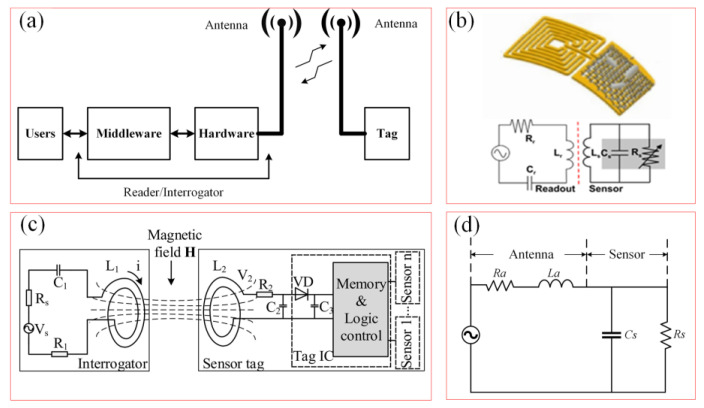
Working principle and system of soft wireless RF sensors. (**a**) The working principles of wireless chipless RFID system. (**b**) Structure diagram of wireless chipless RF sensor [62]. (Copyright (2012) Springer Nature, Berlin, Germany). (**c**) Electromagnetic coupling principle of wireless chipless RF sensor system [54]. (**d**) Equivalent circuit diagram of wireless radio frequency identification sensor.

The working principles of a wireless chipless RFID system are depicted in Figure 4a. This system comprises a tag, an antenna, and a reader/interrogator. The reader incorporates both hardware components for signal transmission and reception, and the middleware is used for data processing and user interaction, mainly including key components such as communication management, signal processing, data encoding and decoding, interface services, error handling, and exception management, as well as security and privacy protection. Figure 4b shows the physical layout and equivalent circuit of a wireless chipless RF sensor, which is composed of resistance, capacitance, inductance, and sensor resistance [62]. Internal impedance without traditional electronic chips responds to changes in external physical quantities, which could be detected by external systems for biological parameter monitoring while offering a low-cost and high-durability solution.

Figure 4c shows an equivalent circuit model of an RFID system [54]. The interrogator generates an alternating magnetic field through the inductor *L*_1_, and activates the circuit in the sensor tag for wireless data transmission detection by changing the resonant frequency, which is suitable for remote, battery-free, and real-time monitoring applications. Figure 4d illustrates the equivalent circuit diagram of a flexible wireless RFID sensor. When external pressure is applied to the sensor, the distance between the ferrite film of the reflective layer and RFID tag decreases. The alteration notably impacts the impedance match between the antenna and the overall tag impedance, subsequently leading to a reduction in the received signal strength indicator (RSSI) of the backscattered signal detected by RFID reader, enabling highly sensitive wireless detection of pressure variations. The impedance of the antenna and RFID tag can be represented as:

(4) $$Z_{a}=R_{A}+jwL_{A}$$

(5) $$Z_{c}=R_{C}/(1+jwC_{C}R_{C})$$

where $Z_{a}$ is the impedance of the tag antenna, $R_{A}$ is the resistance, $jwL_{A}$ is the reactance of the tag antenna, and $R_{C}$ and $1/jwC_{C}$ are the resistance and impedance of the RFID chip. When the external pressure acts on the sensor, the impedance matching between the antenna and the chip changes, affecting the signal gain of the RFID tag, and then the signal strength received by the RFID reader changes. By monitoring the change in signal strength, the pressure applied to the sensor can be inferred. This design allows the RFID system to communicate with the sensor without contact and is suitable for a variety of application scenarios, such as contactless data transmission and energy harvesting.

### 2.3. Soft Wireless Passive SAW Sensors

**Figure 5:**
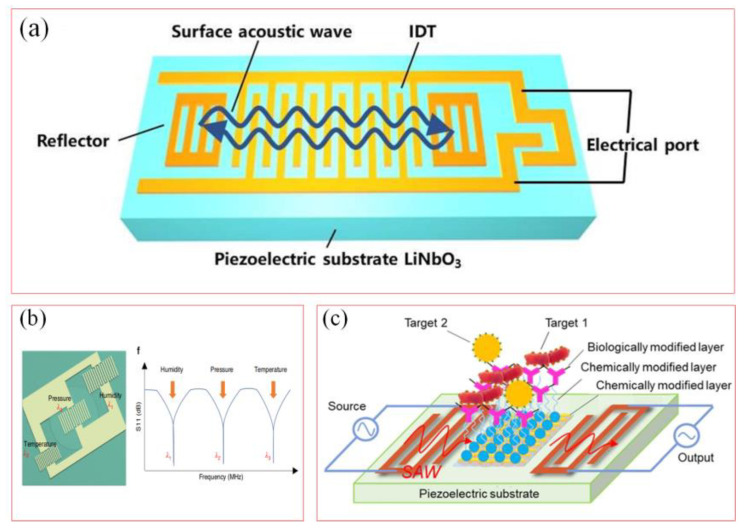
Working principle of soft SAW sensors. (**a**) SAW sensor with IDT and reflective gratings [56]. (Copyright (2018) Elsevier, Amsterdam, The Netherlands). (**b**) Multi-parameter perception principle based on SAW sensors [63]. (Copyright (2023) Springer Nature, Berlin, Germany). (**c**) Basic working principle of SAW biosensor [64]. (Copyright (2024) Royal Society of Chemistry, London, UK).

IDT-SAW sensors are designed with metal patterns in the shape of forks on piezoelectric substrates [14,15]. The electrodes with alternating positive and negative electrodes are arranged periodically on a piezoelectric substrate. High-frequency electrical signals form a potential difference between the two forks of IDT, which generates elastic waves through the inverse piezoelectric effect of the piezoelectric substrate. Figure 5a shows the process of converting SAW into electromagnetic waves involves the reflection of SAW when it encounters a reflective grating on a piezoelectric substrate [56]. The reflected wave is collected by IDT to produce a piezoelectric effect, generating electromagnetic waves along the surface of the piezoelectric substrate.

There are many factors that affect the change in wave velocity. The resonant frequency of surface acoustic wave resonator is related to many parameters such as temperature, humidity, and strain. The relationship between the change in acoustic phase velocity and each parameter is shown in Equation (4). The acoustic signal contains a variety of features, which may be affected by the sensing variables. Therefore, these sensing variables can be measured separately by frequency shift.

(6) $$\frac{\Delta f}{f_{0}}=\frac{\Delta v}{v_{0}}=\frac{1}{v_{0}}(\frac{\partial v}{\partial m}\Delta m+\frac{\partial v}{\partial \sigma }\Delta \sigma +\frac{\partial v}{\partial F}\Delta F+\frac{\partial v}{\partial \varepsilon }\Delta \varepsilon +\frac{\partial v}{\partial T}\Delta T+\frac{\partial v}{\partial c}\Delta c)$$

where Δf is the shift value of the resonance frequency, $f_{0}$ is the initial resonance frequency, Δv is the variable phase velocity of SAW, $v_{0}$ is the wave velocity of SAW sensor, Δm is variable mass, Δσ is the variable conductivity value, ΔF is the variable stress, Δε is the variable dielectric constant, ΔF is the variable pressure, and ΔT is the variable temperature value.

When SAW propagates on the substrate surface, the propagation characteristics (the propagation speed and distance of SAW) are influenced by external factors such as temperature, pressure, magnetic field, electric field, and specific gas. When an SAW sensor is subjected to external excitation, its resonant frequency or delay characteristics will change with the changes in external excitation conditions. Based on the resonant frequency shift, the external sensing parameters are obtained under external excitation conditions.

Combined with structural forms and signal processing circuits, it can be applied to measure mechanical strain, temperature changes, and small displacements. Based on the multi-physical quantity sensing principle of surface acoustic waves, different parameters are sensed at different resonant frequencies. Different resonant units are designed to achieve online perception of different physical quantities for different monitoring object corresponding to different perceived physical quantities (Figure 5b) [63]. Different reflective grating structures are adopted to obtain different echo frequencies corresponding to different physical quantities, to achieve online perception and decoupling of different parameters, and to detect technical indicators. Figure 5c shows the working principles of the surface acoustic wave biosensor [64]. Based on the sensitivity of the surface acoustic wave generated by the piezoelectric substrate, the target biomolecule is specifically bound to the recognition element (such as antibody, enzyme, or nucleic acid probe) on the surface of the sensor, which will lead to the change in the resonance frequency of the SAW. The rapid detection of pathogenic bacteria in food could be achieved by measuring the frequency changes.

## 3. Soft Wireless LC-Resonant Sensors for Biological Monitoring

### 3.1. Soft Wireless LC Strain Sensor with Variable Inductance Value

**Figure 6:**
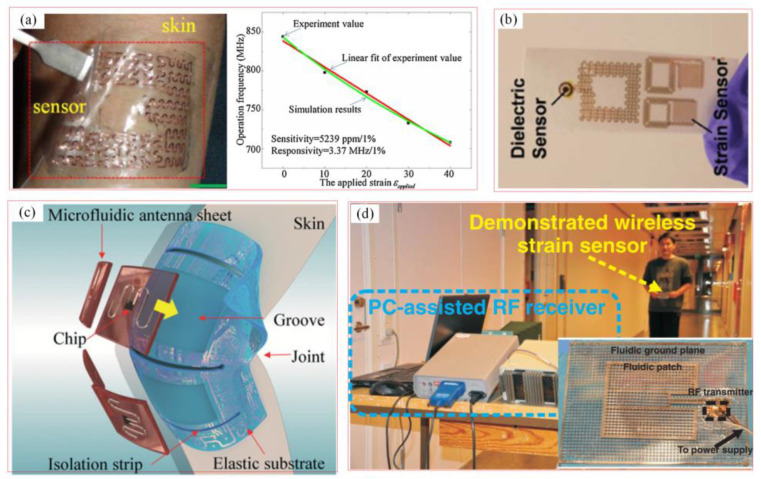
Soft wireless LC-resonant sensors for biological monitoring. (**a**) Stretchable wireless LC strain monitoring with self-similar structure [38]. (Copyright (2015) Elsevier, Amsterdam, The Netherlands). (**b**) Flexible wireless strain and dielectric sensor [53]. (Copyright (2014) WILEY-VCH, Weinheim, Germany). (**c**) Stretchable microfluidic antenna for strain signal and body movement monitoring [65]. (Copyright (2014) Royal Society of Chemistry, London, UK). (**d**) Wireless strain monitoring based on liquid metal antenna [66]. (Copyright (2011) WILEY-VCH, Weinheim, Germany).

Wireless strain sensors based on LC-resonant circuits have been widely used in a variety of sensing fields, such as body surface monitoring and intracranial pressure monitoring. Chipless sensors have attracted much attention because of their excellent anti-interference ability, high sensitivity, better security, and low price. Figure 6a shows a scenario in which a stretchable wireless LC strain sensor with a self-similar structure is applied in the deformation monitoring of a skin surface [19]. The sensors’ scalability enhances their usefulness in detecting structural deformation with the measurement results of return loss in different stretched statuses (from 0 to 40%) [38]. Figure 6b shows a scenario in which a flexible wireless strain sensor is applied to strain and sweat signal monitoring on the human skin [53]. A new type of wireless skin sensor is demonstrated. The dielectric properties and deformation of the skin can be accurately measured by an LC resonator and a capacitive electrode. The experimental results show that the strain detection accuracy is 1.3%. Figure 6c depicts the stretchable microfluidic antenna for strain signal and body movement monitoring [65]. The resonance frequency of the stretchable microfluidic antenna could be modulated by the external strain. Figure 6d shows a scenario in which a wireless strain sensor based on liquid metal antenna is applied to strain monitoring by radio frequency data exchange [66].

### 3.2. Soft Wireless LC Pressure Sensor with Variable Capacitance Value

**Figure 7:**
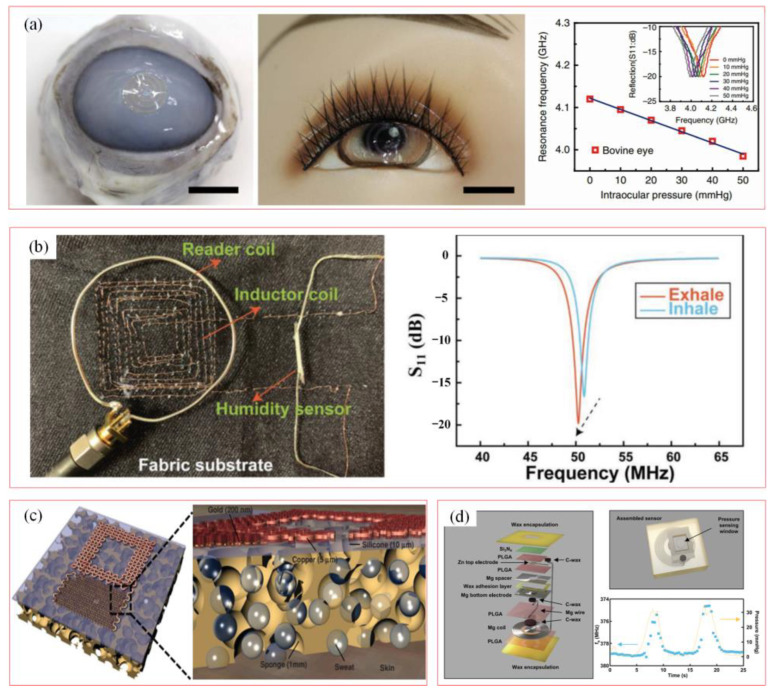
Soft wireless LC-resonant sensors for biological monitoring. (**a**) Pressure measurement with variable capacitance value [67]. (Copyright (2017) Springer Nature, Berlin, Germany). (**b**) Soft strain sensor with variable capacitance value on clothes for biological monitoring [43]. (Copyright (2019) WILEY-VCH, Weinheim, Germany). (**c**) Soft wireless strain and hydration monitoring [68]. (Copyright (2014) WILEY-VCH, Weinheim, Germany). (**d**) Bioabsorbable wireless passive pressure sensor [69]. (Copyright (2020) WILEY-VCH, Weinheim, Germany.)

Flexible wireless passive LC-resonant sensors are applied to pressure monitoring with the variable capacitance value. An LC-resonant circuit with a capacitive sensor is implanted to the anterior chamber for pressure detection. The resonance frequency from soft wireless LC pressure sensor is detected by the antenna reader for devoting the relationship between the pressure and the resonance frequency value. Figure 7a shows the demodulated signal is detected at the analog reader section [67]. Figure 7b depicts a scenario in which soft wireless sensors based on the variable capacitance value are implanted into the clothes for strain monitoring [43]. Soft wireless passive LC sensors are applied onto the human body or embedded in clothes to obtain physiological signals from body movements. Figure 7c depicts a scenario in which a soft wireless LC sensor is designed for multi-parameter perception (temperature, hydration, strain) for more accurate biological healthy monitoring [68]. A wireless sensor is applied to collect hydration and strain information of the skin surface, which is adopted to evaluate the level of hydration of the skin on the forearm. Figure 7d shows an innovative biodegradable wireless pressure sensor as a temporary implant for in vivo pressure measurement (such as intracranial pressure, pulmonary hypertension, etc.) [69]. The designed soft wireless passive LC sensor achieves a sensitivity of up to 200 kHz mmHg^−1^, providing a new solution for medical monitoring without surgical removal. However, due to the limited lifespan of the sensor, which could work stably in vivo for a few days, and it may not be suitable for long-term monitoring applications.

Soft wireless LC sensors with variable inductance and capacitance values have been widely applied in biological monitoring due to their resonance frequency shift. Soft wireless LC sensors without chipped devices could be integrated onto the skin surface conformally; this is because the inductance and capacitance components can be designed with good bendability and stretchability. This would enhance the advantages of soft wireless chipless LC sensors by EM transduction for temperature, pressure, humidity, gas detection, strain, and biosensing monitoring.

## 4. Soft Wireless RF Sensors for Biological Monitoring

RF sensors combine radio frequency identification technology and sensing technology, which is a new research field. Chipless RF sensor is a kind of sensor based on the electromagnetic conduction mechanism, which realizes the remote measurement of physical quantities without any active power supply or signal processing circuit. Wireless RF sensors are receiving increasing attention for research and development in the field of biological applications due to their unique advantages in real-time monitoring, data transmission, and environmental adaptability. As a new type of sensor, wireless and chipless RF sensors utilize radiofrequency signals for data acquisition and transmission, and realize the wireless transmission of data without interfering with living organisms through RF signals for efficient monitoring and data acquisition.

**Figure 8:**
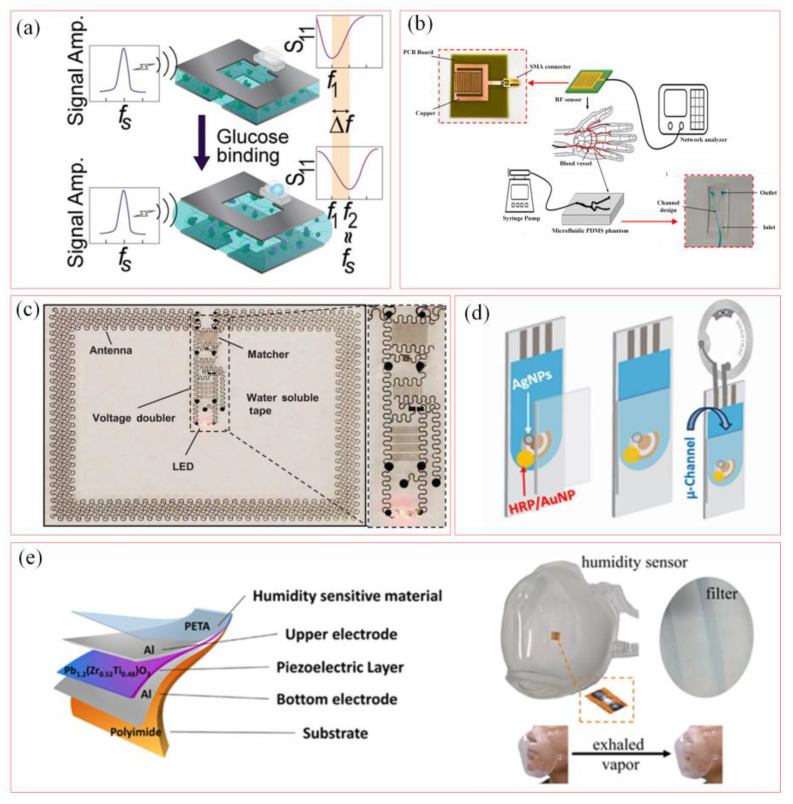
Soft wireless RF sensors for biological monitoring. (**a**) Wireless passive RF sensor for glucose concentration detection [70]. (Copyright (2020) Elsevier, Amsterdam, The Netherlands). (**b**) RF sensor for remote monitoring of blood glucose levels [71]. (**c**) A skin RF electronic device for wireless transmission [55]. (Copyright (2016) Springer Nature, Berlin, Germany). (**d**) Wireless passive biosensor based on RFID tags [72]. (Copyright (2019) Springer Nature, Berlin, Germany). (**e**) Flexible RF sensor for human respiratory humidity monitoring [73]. (Copyright (2024) American Chemical Society, Washington, DC, USA).

Figure 8a shows a wireless passive RF resonator based on phenylboronic acid hydrogel for implantable glucose monitoring biosensors [70]. The resonance frequency of phenylboronic acid hydrogel is adjusted by the response of phenylboronic acid hydrogel to the change in glucose concentration, and the wireless power transmission and light intensity report of glucose concentration are realized with high sensitivity and a fast response to the change in glucose concentration. Figure 8b shows an innovative RF sensor for continuous monitoring of blood glucose levels, which realizes the non-invasive continuous monitoring of blood glucose levels by detecting changes in the resonant frequency of the resonator by the variable dielectric constant of the blood [71]. The performance of the sensor was verified by manufacturing a microfluidic device that simulates the vascular structure of human hands. The sensitivity of the sensor is 264.2 kHz/mg·dL^−1^, which can detect glucose concentration changes that are consistent with human blood glucose levels.

Figure 8c shows a schematic diagram of a modular skin RF system for wireless power transmission [55]. The system achieves energy harvesting through an ultrathin antenna, a rectifier, and a voltage multiplier. The antenna has a gain of 2.89 dB (1.65 GHz) in the air and a gain on the skin decreases to −18.5 dB. At a distance of 1.5 m and a power of 15 W, the system generates an open circuit voltage of about 8 V in the air and 6.5 V on the skin, demonstrating its potential applications in epidermal electronics. Figure 8d presents an innovative wireless passive biosensor based on RFID tags [72]. The design utilizes silver nanoparticles (AgNPs) as part of the RFID antenna for wireless sensing by monitoring biological redox reactions. The sensor could be widely applied to the detection of various biological redox reactions. RFID biosensors have certain limitations in multi-analyte detection and quantitative analysis. Figure 8e presents a soft RF sensor for detecting human respiratory humidity [73]. Using sensitive materials for humidity changes, it has a significant linear correlation between the resonant frequency and the humidity level of 65% to 86%, which is consistent with the typical humidity range of the gas during human respiration. The flexible design is conducive to better integration into wearable devices to achieve continuous real-time monitoring of human respiratory conditions.

## 5. Soft Passive SAW Sensors for Biological Monitoring

### 5.1. Biological Monitoring

**Figure 9:**
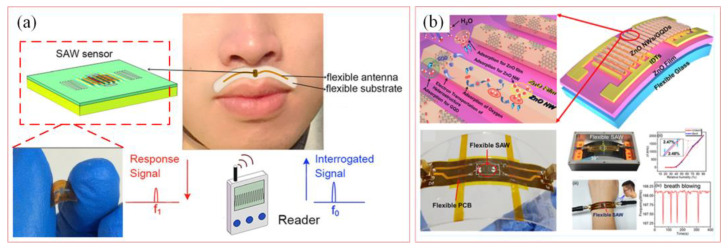
SAW sensor for biological application. (**a**) Schematic illustration of sensing mechanisms of SAW sensor for breath monitoring [75]. (Copyright (2017) IOP Publishing Ltd., Bristol, UK). (**b**) Flexible SAW device packaged with polyimide PCB for biological health monitoring [76]. (Copyright (2020) American Chemical Society, Washington, DC, USA).

SAW sensors are widely applied to wearable electronics, biomedicine, and microfluidics using specific geometries and materials. The geometries are improved, and multi-aperture detection is performed to increase sensitivity and selectivity. Wearable SAW electronics are used to human movement, health, and physiological signal monitoring [13,24,74]. Figure 9a depicts a wireless passive SAW sensor system [75]. By measuring the relationship between the resonant frequency of the SAW sensor and the humidity level, a wireless passive SAW sensor system is proposed for respiratory monitoring. Figure 9b presents a flexible, transparent SAW humidity sensor based on ultrathin glass encapsulated onto a flexible polyimide film printed circuit board, achieving an extremely high humidity sensitivity of 40.16 kHz/%RH [76]. Sensor performance tests at a 30° bending angle show no significant degradation in humidity sensing performance, demonstrating the potential applications on curved and complex surfaces. Sections i, ii, iii, and iv demonstrate the flexibility of the SAW sensor, which can be worn on the wrist and other parts of the body to monitor the humidity changes in the surrounding environment and the breathing process of the human body in real time.

**Figure 10:**
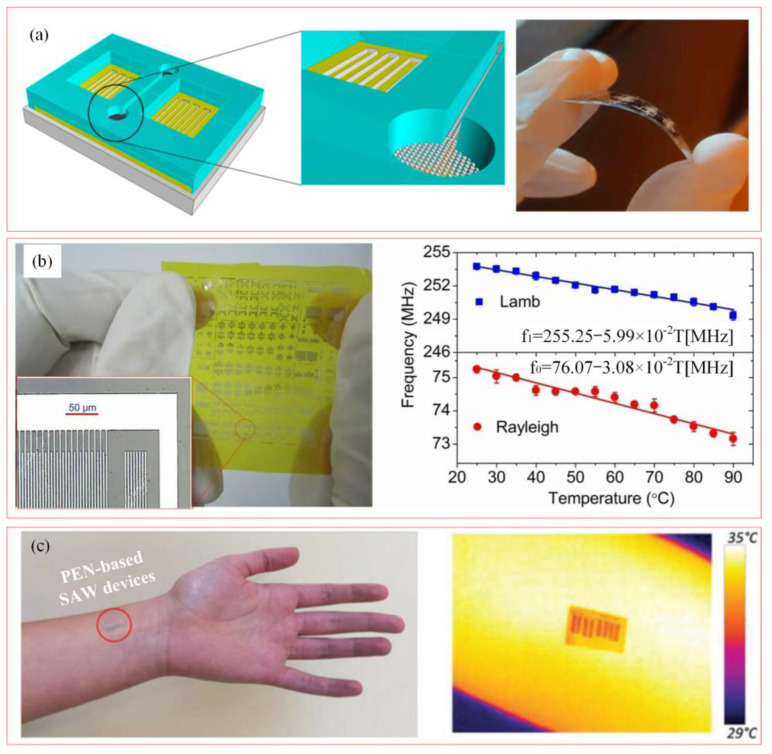
SAW sensors for wearable electronics applications. (**a**) Flexible wearable SAW devices for liquid pH measurement [77]. (**b**) SAW temperature sensor application [78]. (Copyright (2013) Springer Nature, Berlin, Germany). (**c**) Flexible SAW temperature sensor for temperature visualization [57]. (Copyright (2019) WILEY-VCH, Weinheim, Germany).

Figure 10a shows a flexible microfluidic pH sensor based on SAW, which is fabricated on a polyethylene terephthalate (PEN) substrate with good flexibility and uses ZnO nanoparticles (NPs) as the pH-sensitive layer [77]. The resonance frequency of the SAW device is linearly related to pH value. It exhibits a high sensitivity of about 30 kHz/pH in the range of pH 7 to pH 2, and it has broad application prospects in wearable devices and biomedical detection fields. Figure 10b presents the application of SAW temperature sensor in human body temperature measurement [78]. It is necessary to ensure the accuracy of the measured data of the sensor on the curved surface. Designing the appropriate structure and optimizing the thickness and quality of the piezoelectric layer can improve the flexibility and skin adaptability of the device.

Figure 10c shows a novel AIN-based flexible and transparent SAW sensor using PEN as the substrate material [57]. Experiments have shown that the SAW device has an extremely high temperature coefficient of 810 ppm/°C, which has a great potential for application in the field of flexible electronics and wearable technology. However, multi-layer AlN deposition and subsequent micro-machining steps increase the complexity and cost of the manufacturing process, and simpler manufacturing processes can be explored in the future to reduce costs and increase production.

### 5.2. SAW Microfluidics

**Figure 11:**
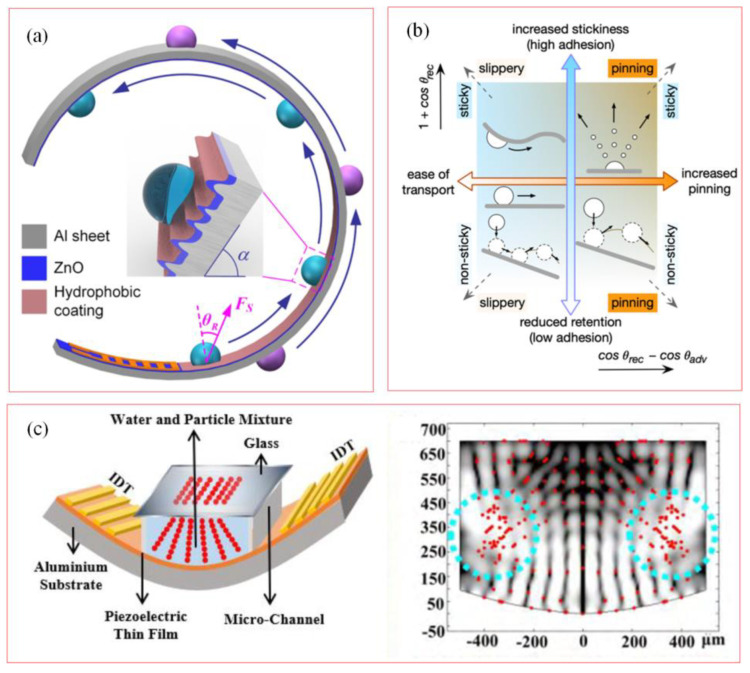
Flexible acoustofluidics for biological monitoring. (**a**) Surface acoustofluidics for flow velocity measurement [79]. (Copyright (2021) American Chemical Society, Washington, DC, USA). (**b**) surface acoustofluidics with ZnO/Al foil [80]. (Copyright (2020) American Chemical Society, Washington, DC, USA). (**c**) Bendable acoustofluidics for microfluidics monitoring [81]. (Copyright (2021) Elsevier, Amsterdam, The Netherlands).

Flexible acoustofluidics with polymers, including PI and PET as the flexible substrates, have been applied in biological health monitoring. However, the acoustic wave dissipation on the flexible substrate is more significant, which seriously affects the performance of the device. Choosing the appropriate flexible substrate material and design is the key to ensuring the effective propagation of acoustic waves. Figure 11a shows the acoustic fluid performance of SAW devices based on zinc oxide (ZnO) thin films on flexible, bendable aluminum foils/sheets with different thicknesses (50–1500 μm), providing the best conditions for realizing acoustic fluid function on bendable and deformable surfaces [79]. However, when aluminum foil is used as the substrate material, due to its poor stiffness, large deformation will occur under high RF power, resulting in significant acoustic energy dissipation, affecting the performance of acoustic fluid, and reducing the efficiency of droplet transport. Figure 11b demonstrates the application of hierarchical nanotexture technology on flexible surfaces, especially how to use acoustic fluidics to achieve precise manipulation of liquids [80]. By depositing and controlling ZnO nanostructures on aluminum foils, it shows the ability to efficiently move liquids in three-dimensional space with inclined, vertical, inverted or flexible geometries, which promotes the development of advanced microfluidic applications. Figure 11c depicts a scenario in which soft bendable acoustofluidics with piezoelectric thin film have been applied in microfluidics monitoring. The effects of bending and twisting SAW devices on the patterning of particles and cells are discussed, as can be seen in the blue circles, where the particles on both sides of the microchamber do not form any patterns, and the distribution of particles has a distinctly random character; soft bendable acoustofluidics with particle alignment are more efficient [81,82].

## 6. Conclusions and Future Perspectives 

The purpose of the present review paper is to outline the applications of soft wireless passive chipless sensors and to provide classification of wireless passive sensors and an overall explanation of the main work. External information is obtained by soft wireless passive sensors via the external coils based on electromagnetic induction. Three kinds of soft wireless sensors are introduced, and their working principles, equitant circuits, and biological applications are described: soft wireless passive LC-resonant sensors, soft wireless RF sensors, and soft wireless SAW sensors. Soft wireless passive sensors with LC-resonant units can be applied in physical quantity measurement for determining the mapping relationship between the frequency resonance and a monitored object. Soft wireless RF sensors analyze the reflection and scattering of RF signals by detecting and measuring changes in electromagnetic characteristics in biological tissues or systems, so as to obtain corresponding state parameter information. SAW devices with piezoelectric substrate are applied in physical parameter monitoring, interacting with the mechanical properties of guided waves in SAW sensors.

The application of soft wireless passive sensors is not limited to the acquisition of measurements via electromagnetic resonance or integration with sensing elements for wireless data transmission. The investigations that can be conducted using these sensors are bidirectional: (1) innovative designs should be adopted using soft wireless passive sensors with sensitive material and circuits; (2) innovative applications for expanding the adaptability of soft wireless passive sensors can be used in structural health monitoring of engineering fields for long-term and large-space monitoring.

**Figure 12:**
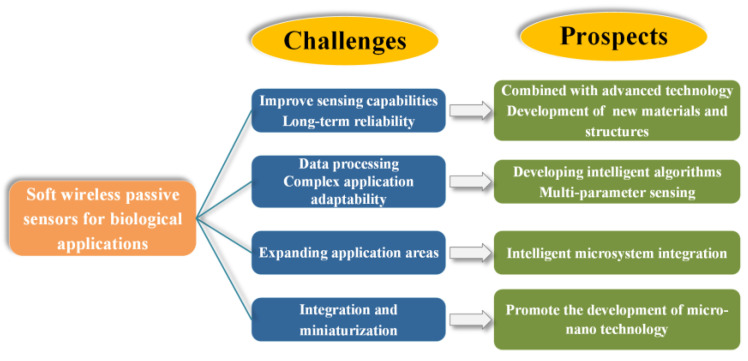
Challenges and prospects of soft wireless passive sensors in biological applications.

The progress of soft wireless passive sensors still has some technical challenges due to stability, as shown in Figure 12. The future perspectives for soft wireless passive sensors are concluded to be the following:

(1) Soft wireless passive sensing technology is a hot research topic. It will be integrated with materials science, microelectronics, and information technology. It can improve sensing functionality through the use of different design methods and material performances. Research and use of new materials, including conductive polymers, two-dimensional materials (such as graphene, MXenes), and nanomaterials, can be implemented to enhance the flexibility, biocompatibility, and sensitivity of the biosensors.
(2) Soft wireless passive sensors should be developed with multi-parameter sensing to adapt more complicated application cases. Intelligent algorithms should be studied to determine the relationship between sensing data with monitoring objects. More sensing data would provide more biological data and accurate disease diagnosis for clinical citation.
(3) Soft wireless passive sensors can be developed integrated with intelligent microsystems. This could enhance the application fields for soft wireless passive sensors, as data sensing, collection, process, and transmission could be realized in intelligent microsystems.
(4) The current production costs of soft wireless passive sensors remain high, especially in materials and manufacturing processes. Determining how costs can be reduced through technological innovation and large-scale production is the key to promoting market acceptance.

## Figures and Tables

**Table 1:** Working principles for soft wireless passive chipless sensors; (soft wireless passive LC-resonant sensor (copyright (2021) IOP Publishing Ltd., Bristol, UK; copyright (2014) WILEY-VCH, Weinheim, Germany); soft wireless RF sensor (schematic graph: copyright (2016) Springer Nature, Berlin, Germany); soft wireless SAW sensor (copyright (2018) Elsevier, Amsterdam, The Netherlands; copyright (2019) WILEY-VCH, Weinheim, Germany).

	Circuit Analysis	Schematic Graph	Principle
Soft wireless passive LC-resonant sensor [36,53]	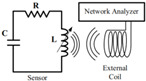	LC-resonant circuit 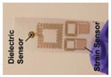	Resonant frequency measurement of LC sensors
Soft wireless RF sensor [54,55]	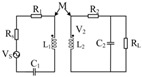	Stretchable RF devices 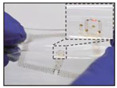	Radio frequency signal frequency measurement of soft RF sensors
Soft wireless SAW sensor [56,57]	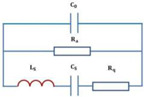	SAW with piezoelectric film 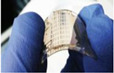	Reflecting frequency measurement of soft SAW sensors
